# Interdisciplinary Surgical Treatments and Long-Term Outcomes of Lumbar Spinal Tumors With Retroperitoneal Involvements: A Retrospective Case Series Study

**DOI:** 10.3389/fonc.2021.720432

**Published:** 2021-12-23

**Authors:** Shaohui He, Yifeng Bi, Chen Ye, Dongyu Peng, Jianru Xiao, Haifeng Wei

**Affiliations:** ^1^ Spinal Tumor Center, Department of Orthopaedic Oncology, No. 905 Hospital of People's Liberation Army (PLA) Navy, Changzheng Hospital, The Second Military Medical University, Shanghai, China; ^2^ Department of Orthopaedic Surgery, Chengdu Military General Hospital, Chengdu, China

**Keywords:** interdisciplinary surgery, lumbar spinal tumor, vascular and urinary involvements, prognosis, *en bloc*, case series

## Abstract

**Purpose:**

Surgical treatments are technically challenging for lumbar spinal tumor (LST) with extensive retroperitoneal involvements. Our study aimed to report the experience and outcomes concerning interdisciplinary surgical collaborations in managing such LSTs.

**Patients and Methods:**

Nine patients underwent interdisciplinary surgical treatments which were performed by specialists, namely, spinal, vascular, and urinary surgeries. Data on clinical characteristics were collected, and the Visual Analogue Scale (VAS) and the Japanese Orthopaedic Association Score (JOAS) were used in the evaluation before and after surgery. The postoperative complications and the long-term outcomes were reported as well.

**Results:**

The interdisciplinary work included double J catheter indwelling (*n* = 9), nephrostomy (*n* = 5), replacement of the common iliac vein (*n* = 2), abdominal aorta repair (*n* = 3), and vital vessel repair (*n* = 8). The early-stage complications included complaints of moderate low back pain and slight implant shift (*n* = 1, 11.1%) and tardive ureterodialysis (*n* = 1, 11.1%). The 3- and 5-year disease-free survival rates were 76.2 ± 14.8 and 50.8 ± 23.0%, respectively, during the mean follow-up of 34.6 ± 17.9 months (range, 9.5–68.7). Besides this, more blood loss was associated with recurrent and metastatic tumor status (*p* = 0.043) and surgery time >5 h (*p* = 0.023). Remarkable pain relief and favorable quality of life were achieved based on the postoperative VAS (3.3 ± 0.9, *p* < 0.001) and JOAS (16.6 ± 0.5, *p* < 0.001).

**Conclusions:**

The treatments of LSTs with wide-range retroperitoneal involvements require interdisciplinary surgical collaborations to lower the risks and improve the long-term outcomes. High-quality prospective cohort studies with large samples are warranted to establish general surgical protocols in managing LSTs with extensive retroperitoneal involvements.

## Introduction

Lumbar spinal tumors (LSTs) are usually discovered with a considerably large size at initial diagnosis due to the retroperitoneal space ahead of the vertebra (1, 2). The tumor grows gradually in the retroperitoneum and compresses or infiltrates the adjacent normal tissue, including vital vascular structures, kidney, and ureter (2, 3). The giant spinal tumors usually arise from the lumbosacral area with a relatively rare incidence (4–6). It was reported that only 20% of patients presented with significant neurologic deficits, while the remaining symptoms were non-specific (7). The most common pathology of giant spinal tumors is giant invasive spinal schwannoma, which accounts for nearly 1/3 of all primary intra-spinal tumors (8). The adjacent vertebral body and neurovascular structures are usually eroded by the invasive tumor biology. Moreover, it is quite common that over 5 years are required to achieve a confirmed diagnosis after symptom onset with a lumbosacral mass (9).

It remains technically challenging to manage LST patients with extensive retroperitoneal involvements, which often require multidisciplinary cooperation to achieve a tumor-free margin and reduce the incidence rate of iatrogenic injury of the vascular and urinary systems. To achieve long-term favorable outcomes, this study herein aimed at reporting the interdisciplinary surgical treatments for such patients, mainly including *en bloc* tumor resection, reservation of urinary function, and repair/replacement of vascular structures.

## Patients and Methods

### Baseline Information and Clinical Findings

The study design was a retrospective, single-center case series study. Nine consecutive patients with LSTs were referred to our institution, which was an affiliated research and teaching hospital of a medical university. Each had a confirmed diagnosis of giant LSTs and extensive involvements of vital vascular and/or urinary structures. For each patient, the tissue or organs in the retroperitoneum (*e*.*g*., vital artery and vein, kidney, and ureter) were compressed or infiltrated by tumor. The exclusion criteria included spinal tumors not located in the lumbar spine, skip malignancy across the spinal column, distant metastases at initial diagnosis, and lumbar spinal tumor without severe adjacent tissue/organ involvements. The clinicopathological information of these nine patients was collected and analyzed comprehensively.

### Diagnostic Assessment

**Figure 1:**
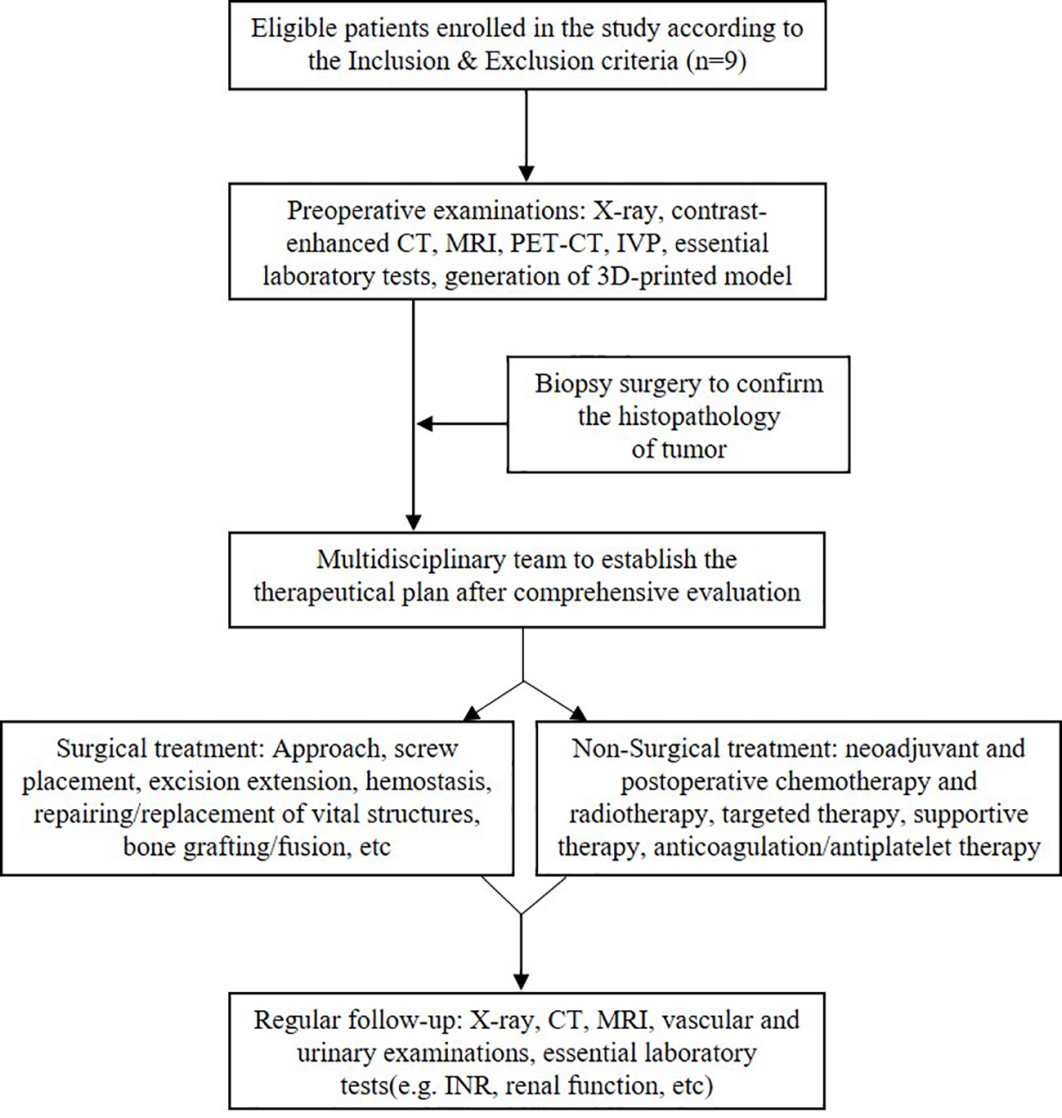
Flow chart of the therapeutic strategy in managing lumbar spinal tumor patients with extensive retroperitoneal involvements.

**Figure 2:**
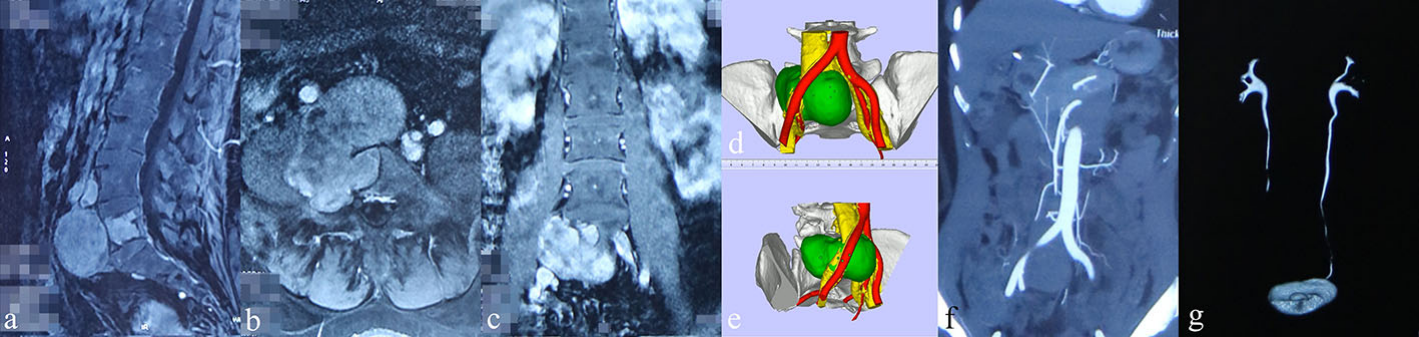
Preoperative images of radiological examinations and 3D-printed models (patient #1). **(A–C)** Sagittal, coronal, and transverse contrast-enhanced MRI indicated moderate intensity of the tumor mass with adjacent invasion, respectively. **(D, E)** 3D-printed model displaying the relation between tumor (green), artery (red), and vein (yellow). **(F)** CT angiography showing the obvious compression of the right common iliac artery by the tumor mass. **(G)** The intravenous pyelography revealed an obstruction of the urinary flow in the right ureter.

As illustrated in **Figure 1**, all patients received contrast-enhanced computed tomography and magnetic resonance imaging routinely. Positron emission tomography–computed tomography was performed, if necessary, to detect potential metastases. Intravenous pyelography (IVP) was conducted to examine the urinary system. Meanwhile, three-dimensional (3D) printing technique was utilized to establish a 3D printed model (1:1) to assess the relationship between the tumor and the adjacent structures (**Figure 2**). Besides this, pre-intervention optimization was conducted to exclude contraindications (*e*.*g*., medication review, treating hypertension and/or diabetes). The potential benefits as well as risks regarding the interdisciplinary surgical procedures were introduced to the patients and their family members.

### Therapeutic Intervention

**Figure 3:**
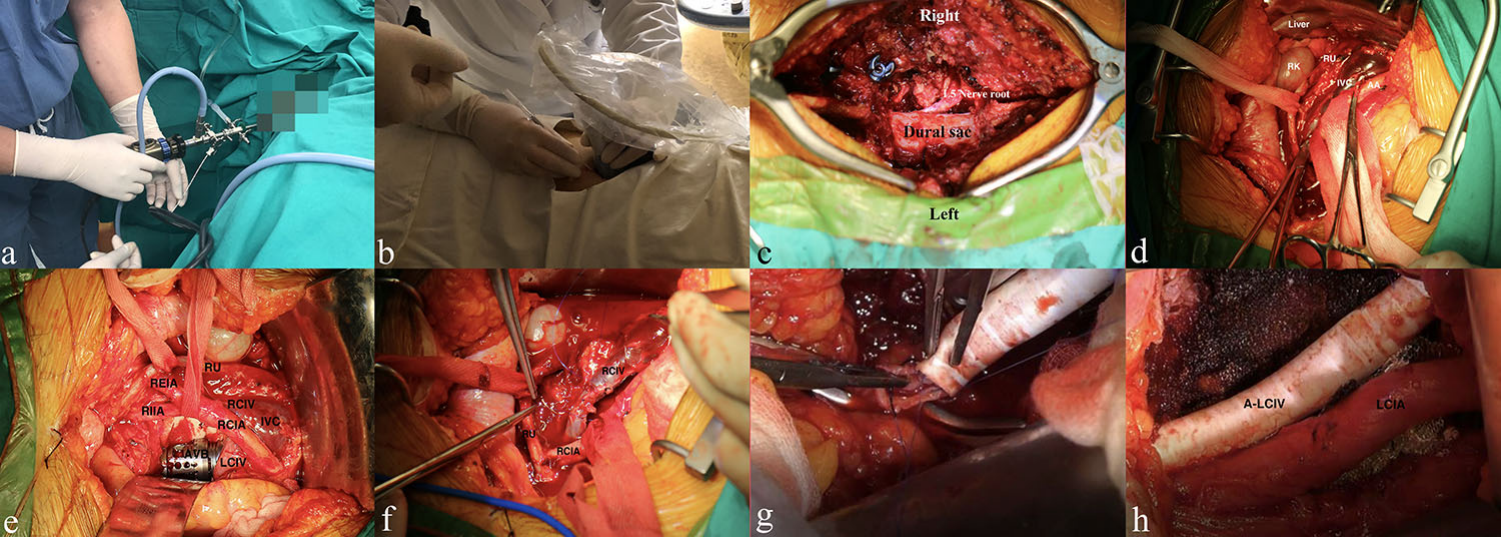
Management of the urinary system, lumbar spine tumor, and vascular condition from the multidisciplinary team. **(A, B)** Double J catheter indwelling and percutaneous nephrostomy for lumbar spinal tumor patients with renal dysfunction and/or ureteral compression, respectively. **(C)** Placement of the pedicle screws, decompressive laminectomy, and identification of the right L5 nerve root and dural sac. **(D)** Blunt separation and protection of the ureter, vascular structure, and vital organs. **(E)** Surgical repair of an infiltrated vein by using atraumatic suture (5-0 Prolene, Johnson & Johnson, USA). **(F)** Placement of the artificial vertebral body (AVB) and identification of adjacent significant structures. **(G, H)** Replacement of the left common iliac vein with a biological substitute. RK/LK, right/left kidney; RU, right ureter; IVC, inferior vena cava; AA, abdominal aorta; RCIA, right common iliac artery; RIIA/REIA, right internal/external iliac artery; RCIV/LCIV, right/left common iliac vein; LRA/RRA, left/right renal artery; LRV/RRV, left/right renal vein; AVB, artificial vertebral body; A-LCIV, artificial left common iliac vein.

All patients with primary tumors received biopsy at initial diagnosis to confirm the tumor histology and underwent staged surgical procedures within 7 days after the confirmed diagnosis. Personalized surgical strategies were generated based on the involved segments and destructive area and the tumor pathology. Double J catheter indwelling was initially performed through the ureteroscope by the urologists if the IVP indicated ureter stenosis (**Figure 3A**), while percutaneous nephrostomy was conducted under ultrasound guidance by the urologists for patients with severe hydronephrosis (**Figure 3B**).

With regard to spine reconstruction, each patient was evaluated initially by using the Spinal Instability Neoplastic Score (SINS) (10). The referred parameters included the tumor location, pain, bone lesion, radiologic spinal alignment, vertebral body collapse, posterolateral involvement, and spinal elements as well as the willingness of the patient to undergo reconstruction surgery. Stabilizing procedures would be performed if the total score ≥13 (the mean SINS was 15.1 ± 1.1, **Table 1**). Every operation was mainly performed by specialists, *i*.*e*., spinal tumor surgery, vascular surgery, and urology. They had rich experience in the referred field, with at least 10 years of training after graduation. Briefly, for the posterior operation, regular exposure, laminectomy, and facetectomy were performed with pedicle-screw rod instrumentation (screw diameter/length: 6.0–6.5 mm/45 mm; rod diameter: 5.5 mm) through a mid-posterior approach (Depuy Synthes, USA). A unilateral titanium rod was fixed transiently for gentle blunt dissection between the ventral dura and the posterior vertebral body wall. The lumbar nerve roots were identified and separated softly to remove the compression (**Figure 3C**). If possible, the tumor was then removed in an *en bloc* fashion through a posterior-only approach. Proper compression was exerted on the fixation system to maintain lumbar lordosis.

**Table 1:** Clinical characteristics and tumor involvements of nine patients.

No.	Age/sex	DOS (M)	Tumor level	Tumor status	Pre. VAS	Pre. JOAS	SINS	Adjacent involvements
1	58/F	0.5	L5	Primary	8	6	15	RCIA, RCIV, RU
2	27/M	60.0	L3–5	Recurrent	6	8	16	RCIA, RCIV, RU
3	55/F	13.0	L5	Recurrent	8	8	14	LCIV, RCIV
4	28/M	1.0	L1–2	Primary	9	7	14	LK, LRA, LRV, LU
5	29/F	24.0	T12–L2	Recurrent	9	8	17	RK, RRA, RRV, RU
6	29/M	3.5	L1–2	Recurrent	8	7	15	RK, RRA, RRV, RU
7	57/M	7.0	L5	Primary	9	7	15	LU, LCIA, LCIV,
8	32/M	9.0	L3–5	Metastatic^a^	9	8	16	LU, LCIA, LCIV, LK
9	66/M	3.0	L3	Primary	9	7	14	RK, RRA, RRV, RU

DOS, duration of symptom; Pre., preoperative; VAS, Visual Analogue Scale; FC, Frankel grade classification; JOAS, Japanese Orthopaedic Association Score; SINS, spinal instability neoplastic score; RCIA, right common iliac artery; RCIV, right common iliac vein; RU, right ureter; LCIV, left common iliac vein; LK, left kidney; LRA, left renal artery; LRV, left renal vein; LU, left ureter; RK, right kidney; RRA, right renal artery; RRV, right renal vein.

a Directly spreading from the retroperitoneal malignant fibroblastoma.

Otherwise, the patient was transferred to the supine position, and an anterior para-median longitudinal incision was performed at 1 to 2 cm from Hunter’s line according to the tumor location and size. The post-peritoneum was opened after layer-by-layer exposure for further detection and separation. During the detection, the vital organs and structures, including the kidney, abdominal aorta, bladder, and uterus, were identified and protected carefully (**Figure 3D**). Gentle traction, if necessary, was performed to achieve a sufficient surgical field. The infiltrated common iliac vein and the involved area of the abdominal aorta were either repaired by sutures or replaced using an artificial biological substitute by the specialized vascular surgeons (**Figures 3D, F–H**). Briefly, for vessel replacement, both ends of the vessel were blocked by smooth forceps every 30 min, and biocompatible substitutes were utilized to reconstruct the vascular continuity, while for vessel or arterial repair, 5-0 Prolene suture (Johnson & Johnson, USA) was used to recover the vascular integrity. The segmental vessels were ligated or coagulated with bipolar forceps, respectively. Then, the tumor mass was isolated using surgical scissors and high-frequency electrotome discreetly, and ultrasonic bone scalpel (XD860A ultrasonic osteotomy system, SMTP Technology Company, China) was used for osteotomy, with at least 0.5-cm distance from the tumor mass. Curette and pituitary rongeur were used to remove the superior and inferior residual intervertebral discs until reaching the normal osseous tissue. Meanwhile, intraoperative frozen pathology was routinely conducted to confirm the tumor-free surgical margins. Prior to this, the artificial vertebra body (AVB) was filled with sufficient allograft bone. The AVB was properly placed after tumor resection, with confirmation of the right position, to reconstruct the spinal continuity and stability (**Figure 3E**). The patients were finally transferred to the intensive care unit after anesthesia recovery for at least 24 h of rigorous monitoring. The patients who underwent vascular replacement/repair received regular double-antiplatelet therapy postoperatively (aspirin, 100 mg, qd; Plavix, 75 mg, qd). Notably, for patients with arterial replacement of the aorta branches, anticoagulation and single-antiplatelet therapy (*e*.*g*., warfarin, 5 mg, tid; Plavix, 75 mg, qd) were recommended to maintain vascular homeostasis, which was monitored using the international normalized ratio (INR = 2.0–3.0).

### Follow-Up and Outcomes

Disease-free survival (DFS) was defined as the interval from the first day after the surgery to the date of tumor-related death, disease progression, or May 31, 2021. The postoperative complications were defined as those which were associated with the disease and the therapeutic modalities after surgery. The Visual Analogue Scale (VAS) was used in the evaluation preoperatively and at 1 month after operation. The Japanese Orthopaedic Association Score (JOAS) (11) was also used to assess each patient before and 3 months after surgery, respectively. The improvement rate of lumbar spine function was calculated by evaluating the postoperative and preoperative JOAS ([Postop. - Preop.]/[29-Preop.] * 100%).

All patients were followed every month for the first 3 months and thereafter every 3 months for the next 12 months through telephone consultation and/or outpatient means. Data on essential clinical condition, blood tests, and radiographic examinations were obtained to evaluate the prognosis. The categorical variables were described by counts and percentages, while the continuous variables were described by mean and standard deviation. Statistics were performed using Student’s *t*-test, and the DFS rate was estimated *via* the Kaplan–Meier method. This case series has been reported in line with the PROCESS Guideline (12).

## Results

Nine consecutive patients with LSTs were enrolled in our study, and the mean age was 42.3 ± 16.1 years old (range, 27–66 years). The main complaints included persistent low back pain and radiating pain with/without dysuresia. Four (44.4%) patients had a recurrent tumor status, while one (11.1%) developed lumbar spinal metastasis which was directly spread from the retroperitoneal malignant fibroblastoma. As depicted in **Table 1**, the duration of symptoms for the patient cohort varies from half a month to 60 months. Three (33.3%) patients were found with 3-segmental tumor involvements and two (22.2%) with 2-segmental invasion. The chemotherapy regimens included adriamycin–ifosfamide for patient #3, vincristine–ifosfamide–etoposide–doxorubicin for patients #4 and #6, and vincristine–adriamycin–cyclophosphamide for patient #7. With regard to radiation, it mainly referred to external beam radiation with 40–50 and 20–25 Gy for patients #5 and #8, respectively. The mean preoperative VAS and JOAS were 8.3 ± 1.0 and 7.3 ± 0.7, respectively. The involved significant tissue and organs included the abdominal aorta, common iliac artery and common iliac vein (CIV), ureter, kidney, renal artery, and renal vein.

For interdisciplinary work, double J catheter indwelling was performed for nine (100.0%) patients, nephrostomy for five (55.6%), artificial CIV replacements for two (22.2%), and abdominal aorta and vital vessel repair for three (33.3%) and eight (88.9%) patients, respectively (**Table 2**). The mean surgery time was 6.0 ± 1.4 h (range, 4.0–8.0), with an estimated average blood loss of 1,689 ± 1,341 ml (range, 600–4,500). Notably, recurrent/metastatic tumor status (*p* = 0.043) and surgery time >5 h (*p* = 0.024) were associated with more blood loss (**Table 3**). The pathological reports revealed benign schwannoma (#1), ganglioneuroma (#2), leiomyosarcoma (#3), Ewing’s sarcoma (#4 and #6), aggressive giant cell tumor (#5), rhabdomyosarcoma (#7), malignant fibroblastoma (#8), and chordoma (#9), all with pathologically confirmed negative surgical margin. Decreased VAS (3.3 ± 0.9) was found after surgery, with statistical significance (*p* < 0.001), and a remarkably improved quality of life (improvement rate: 77.7% ± 9.3%; range 66.6–91.6%) was achieved based on the postoperative JOAS (16.6 ± 0.5, *p* < 0.001).

**Table 2:** Interdisciplinary surgical and non-surgical treatments of nine patients.

No.	Interdisciplinary work	Sur. time (h)	Blood loss (ml)	Tumor pathology	Tumor origin	Adjuvant treatments^a^
1	Double J tube indwelling, nephrostomy, vessel repair	4.5	600	Schwannoma (benign)	Nerve sheath	None
2	Double J tube indwelling, nephrostomy, vessel repair	4	600	Ganglioneuroma (benign)	Ganglion	None
3	CIV replacement, double J tube indwelling, vessel repair	6.5	2,500	Leiomyosarcoma (moderate-well differentiated, grade 3, stage IIB)	Bone	Neoadj. Chemo.
4	Double J tube indwelling, vessel repair	5.0	1,100	Ewing’s sarcoma (dedifferentiated, grade 3, stage IIB)	Bone	Neoadj. Chemo.
5	RK isolation, abdominal aorta and vessel repair, double J tube indwelling	7.5	4,500	Aggressive GCT (moderate–well-differentiated, grade 2, stage IIA)	Bone	Denosumab radiotherapy
6	RK, RRA/V separation, double J tube indwelling, nephrostomy, vessel repair	8.0	2,400	Ewing’s sarcoma (poorly differentiated, grade 3, stage IIB)	Bone	Neoadj. and Postop. Chemo.
7	Double J tube indwelling, abdominal aorta repair, LCIV replacement	5.0	700	Rhabdomyosarcoma (moderate differentiated, grade 2, stage IIA)	Muscle	Neoadj. Chemo.
8	Double J tube indwelling, nephrostomy, LCIV repair	7.0	2,300	Malignant, fibroblastoma (poorly differentiated, grade 3, stage III)	Muscle	Radiation
9	Double J tube indwelling, nephrostomy, abdominal aorta, and RCIV repair	6.5	1,000	Chordoma (poorly differentiated, grade 2, stage IIA)	Notochordal tissue	None

Sur., surgical; Postop., postoperative; LBP, low back pain; GCT, giant cell tumor; LCIV/RCIV, left/right common iliac vein; RK, right kidney; Neoadj., neoadjuvant; Chemo., chemotherapy.

a The details of chemotherapy and radiotherapy could be found in the text.

**Table 3:** Analysis of the clinical factors and outcomes of nine patients.

Factors	Mean ± SD	*p*-value^a^
Blood loss (ml): primary/non-primary	725 ± 125/2,460 ± 1,383	0.043
Blood loss (ml): surgery time ≤5/>5 h	583.3 ± 204.1/558.3 ± 400.5	0.024
VAS: preop./postop.	8.1 ± 1.0/3.3 ± 0.9	<0.001
JOAS: preop./postop.	7.3 ± 0.7/16.6 ± 0.5	<0.001

a Student’s t-test for independent samples.

**Figure 4:**
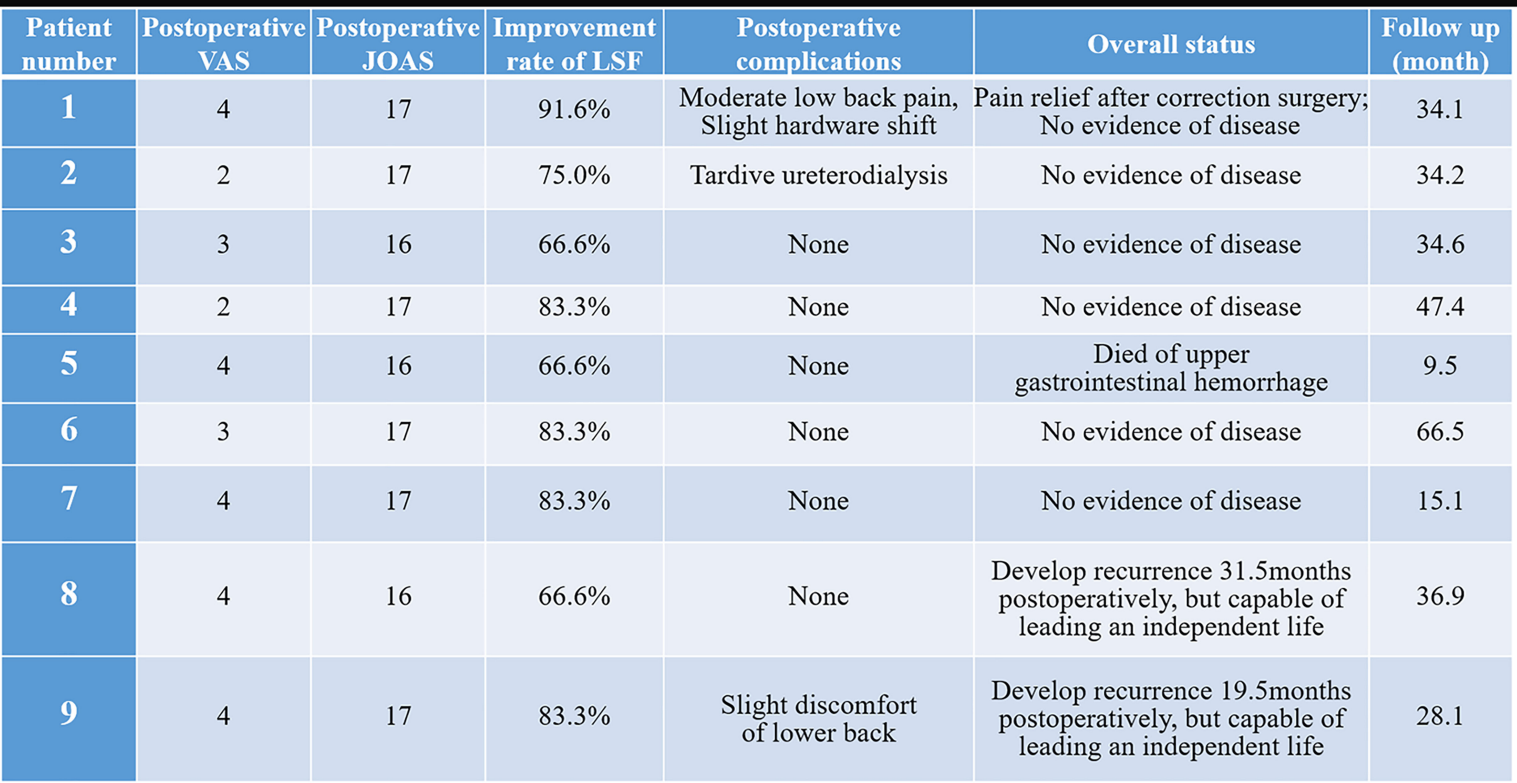
The outcomes of lumbar spinal tumor patients with extensive retroperitoneal involvements. VAS, Visual Analogue scale; JOAS, Japanese Orthopaedic Association Score; LSF, lumbar spine function.

**Figure 5:**
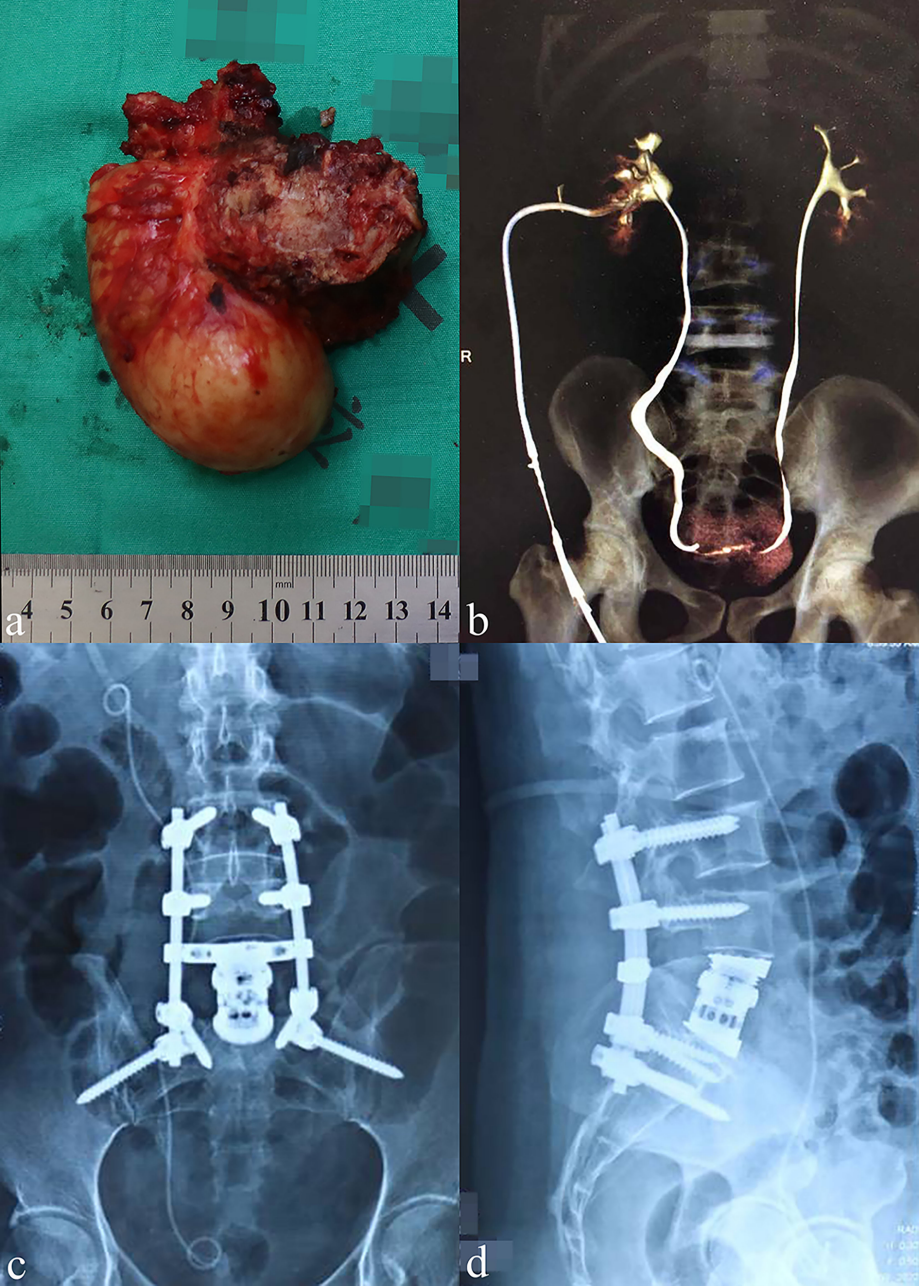
General view of the resected tumor and postoperative examinations. **(A)** General view showing a representative resected tumor (18 × 9 cm) in an *en bloc* fashion. **(B)** The postoperative intravenous pyelography indicated a continuous urinary flow on both sides of the ureters. **(C, D)** The anteroposterior and lateral X-ray of two patients at 24 months post-operatively.

As shown in the chart (**Figure 4**), for the early-stage postoperative complications, one patient (#1, 11.1%) complained of moderate low back pain postoperatively, and the prompt X-ray indicated slight AVB migration. The patient received correction surgery and obtained pain relief later. Another patient (#2, 11.1%) underwent percutaneous nephrostomy since the postoperative IVP revealed hydronephrosis and urine leakage, which was caused by the tardive ureterodialysis. A typical *en bloc* resection of tumor mass is shown in **Figure 5A**, and the postoperative radiological examinations indicated good position of hardware and continuous urine flow (**Figures 5B–D**). During the mean follow-up of 34.6 ± 17.9 months (range, 9.5–68.7), no bleeding/thrombus or other vascular-related issues were observed for patients receiving vessel repair or replacements, and the coagulation indicators also met the expectation (INR = 2.0–3.0) without hemorrhage tendency. In addition, all patients with preoperative urinary involvements got rid of dysuresia and regained normal renal function 1 month after the surgeries.

After recovering spine continuity and stability, the mean improvement rate of lumbar spine function was 77.3% ± 9.3. Eight (88.9%) patients led an independent life with full capacities, among which six (75.0%) had no evidence of disease, while two (25.0%) developed tumor progression. However, these two patients with tumor recurrence had no severe complaints and had a satisfactory quality of life, which was recognized as “alive with disease”. Besides this, one patient (#5, 11.1%) died of disease 9.5 months after the operation. The cause of death was related to upper gastrointestinal hemorrhage which might have been caused by glucocorticoid use and the long-term anti-platelet/coagulation therapy. According to Kaplan–Meier estimation, the 3- and 5-year DFS rates of the patient cohort were 76.2 ± 14.8 and 50.8 ± 23.0%, respectively.

## Discussion

**Figure 6:**
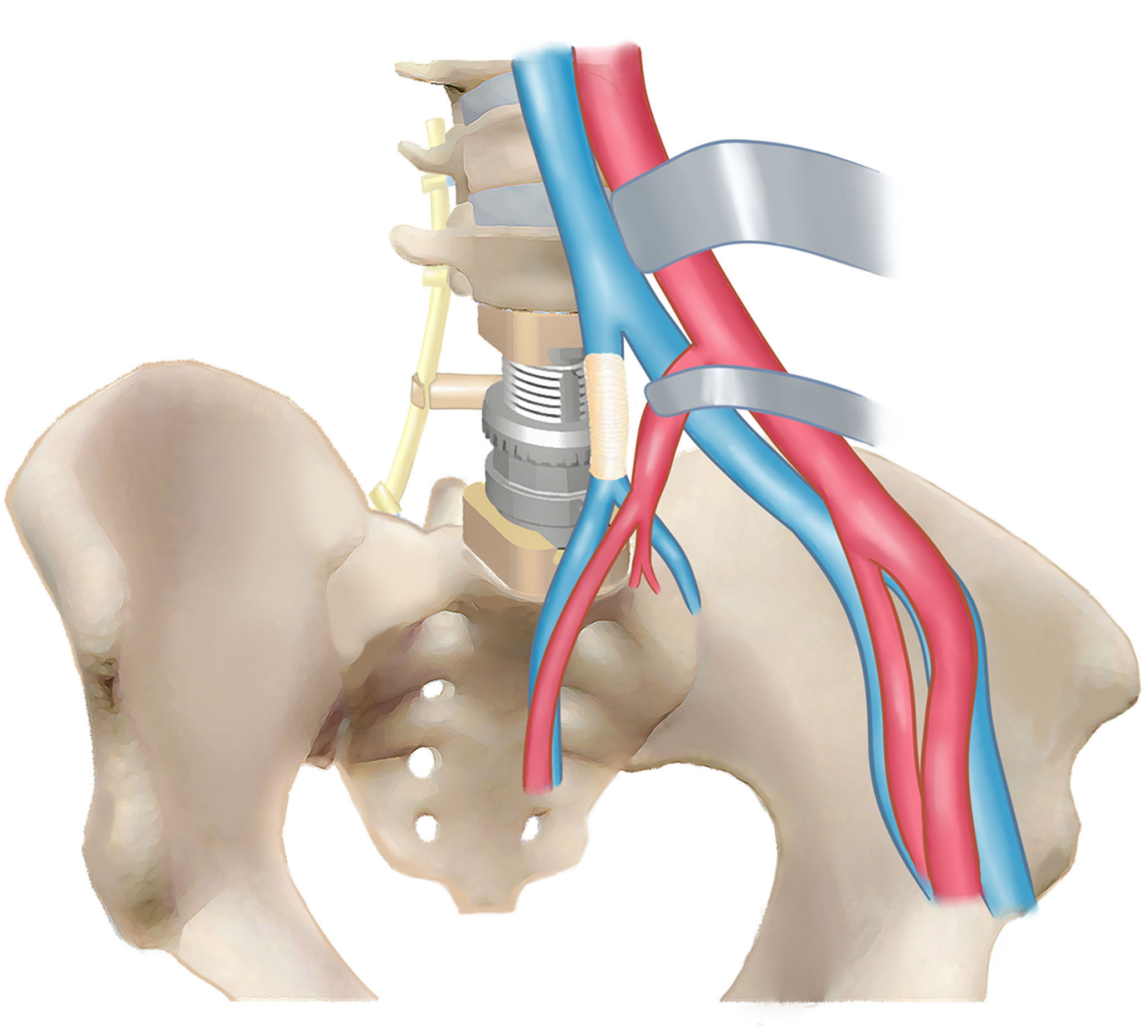
Schematic image of the interdisciplinary surgical collaborations.

The LSTs can be extremely large with significant tissue/organ involvements at initial diagnosis due to the great retroperitoneal space (1–3). Moreover, the diagnosis of LST can be incidental findings for some asymptomatic patients. However, in our study, four (44.4%) patients had back pain with neurologic dysfunction with different extents, while another five (55.6%) had tumor-related surgeries previously. For such giant LSTs with retroperitoneal involvements, surgical management is technically demanding and with high risks because of the complicated neurovascular or other vital organ/tissue infiltration, which always requires multidisciplinary collaboration (**Figure 6**).

The specialized vascular surgeons were invited to manage the vascular bypass, ligation, resection, and replacements. The technical process of combined vessel and spinal tumor resection with reconstruction had been reported feasible and effective in managing giant thoracic spinal tumors (13–15). In our study, a safe resection with tumor-free margin cannot be performed without vascular repair or replacement due to the tumor circumferential growth pattern. To our knowledge, despite numerous studies reporting the surgical management of giant lumbar spinal tumors, no vessel replacement was reported in their operations (16–18). Indeed vascular injury through anterior exposure sometimes is inevitable due to the unclear boundary caused by the aggressive growth of the tumor, and the occurrence of vascular injury is an intractable problem which can be life-threatening (19). Moreover, the morbidity can be up to 3 to 5 times in revision lumbar surgery in contrast with primary ones (20). Since the long-term anticoagulation therapy after bio-prosthesis implantation increased the bleeding risk (21), coagulation parameters should be rigorously monitored to evaluate the outcomes and complications in the long-term follow-up. In our study, two patients received artificial vessel replacements, and they both had favorable coagulation function which was indicated by the INR (2.0–3.0). Notably, the high risk of delayed upper gastrointestinal hemorrhage after lumbar spine surgery should be attended to, and the risk was reported to increase with the long-term use of steroids (22), occurrence of substantial blood loss, fluid/electrolyte disorders, and weight loss (23).

The urologists were invited to perform double J catheter indwelling and percutaneous nephrostomy before spine surgery for LST patients with indications, while the specialist on kidney transplantation was committed to isolating and removing the tumor-infiltrated kidney. The ureter is proximal to the lumbar vertebral bodies which are located in the lateral orientation of the psoas major muscle (24). The routine use of the double J catheter can decrease the urinary-associated complications in the anterior surgical procedures (25, 26) since it can be observed under direct vision or indicated by the intraoperative X-ray machine. Indeed postoperative ureter injury and urinary retention are common in lumbar spine surgery (20, 27, 28). Notably, if the ureter is compressed or even infiltrated in the lumbar spinal tumor, the double J catheter should be placed ahead of the spine surgery to relieve dysuresia and avoid urinary complications intraoperatively.

### Limitations

Although this was the first-reported study focusing on the multidisciplinary treatments of LST patients with extensive retroperitoneal involvements, limitations did exist due to the small sample size and retrospective design. Prospective cohort and longer follow-up are required to establish optimal interdisciplinary therapeutic protocols for better outcomes.

## Conclusions

Interdisciplinary surgical collaborations are required to manage lumbar spinal tumors with retroperitoneal vascular and urinary involvements. Optimal scheme interdisciplinary technical procedures are warranted to reduce the perioperative complication rate and improve the long-term prognosis.

## Data Availability Statement

The original contributions presented in the study are included in the article/**Supplementary Material**. Further inquiries can be directed to the corresponding authors.

## Ethics Statement

The studies involving human participants were reviewed and approved by the institutional review board of Changzheng Hospital, Second Military Medical University. The patients/participants provided their written informed consent to participate in this study. Written informed consent was obtained from the individual(s) for the publication of any potentially identifiable images or data included in this article.

## Author Contributions

HW, JX, and HS designed the study. YB and CY collected the data. HS and DP did the statistical analysis. HW and JX supervised the manuscript and approved the final version. All authors contributed to the article and approved the submitted version.

## Funding

This work was supported by a grant from the National Natural Science Foundation of China (82072971, Haifeng Wei) and Shanghai Science and Technology Committee Program (17411950301, Jianru Xiao). The funding source had no role in the study design, data gathering, analysis, and interpretation, writing of the report, or the decision to submit the report for publication.

## Conflict of Interest

The authors declare that the research was conducted in the absence of any commercial or financial relationships that could be construed as a potential conflict of interest.

## Publisher’s Note

All claims expressed in this article are solely those of the authors and do not necessarily represent those of their affiliated organizations, or those of the publisher, the editors and the reviewers. Any product that may be evaluated in this article, or claim that may be made by its manufacturer, is not guaranteed or endorsed by the publisher.

## References

[1] Kratzig T Dreimann M Klingenhofer M Floeth FW Krajewski K Eicker SO. Treatment of Large Thoracic and Lumbar Paraspinal Schwannoma. Acta Neurochir (Wien) (2015) 157(3):531–8. doi: 10.1007/s00701-014-2320-5 25577451

[2] Messiou C Morosi C. Imaging in Retroperitoneal Soft Tissue Sarcoma. J Surg Oncol (2018) 117(1):25–32. doi: 10.1002/jso.24891 29193092 PMC5836919

[3] Messiou C Moskovic E Vanel D Morosi C Benchimol R Strauss D. Primary Retroperitoneal Soft Tissue Sarcoma: Imaging Appearances, Pitfalls and Diagnostic Algorithm. Eur J Surg Oncol (2017) 43(7):1191–8. doi: 10.1016/j.ejso.2016.10.032 28057392

[4] Yu D Choi JH Jeon I. Giant Intradural Plexiform Schwannoma of the Lumbosacral Spine - A Case Report and Literature Review. BMC Musculoskelet Disord (2020) 21(1):454. doi: 10.1186/s12891-020-03492-y 32652976 PMC7354678

[5] Yu NH Lee SE Jahng TA Chung CK. Giant Invasive Spinal Schwannoma: Its Clinical Features and Surgical Management. Neurosurgery (2012) 71(1):58–66. doi: 10.1227/NEU.0b013e31824f4f96 22353794

[6] Sridhar K Ramamurthi R Vasudevan MC Ramamurthi B. Giant Invasive Spinal Schwannomas: Definition and Surgical Management. J Neurosurg (2001) 94(2 Suppl):210–5. doi: 10.3171/spi.2001.94.2.0210 11302622

[7] Lee MT Panbehchi S Sinha P Rao J Chiverton N Ivanov M. Giant Spinal Nerve Sheath Tumours - Surgical Challenges: Case Series and Literature Review. Br J Neurosurg (2019) 33(5):541–9. doi: 10.1080/02688697.2019.1567678 30836023

[8] Paulo D Semonche A Tyagi R. Surgical Management of Lumbosacral Giant Invasive Spinal Schwannoma: A Case Report and Literature Review. World Neurosurg (2018) 114:13–21. doi: 10.1016/j.wneu.2018.02.146 29510280

[9] Payer M. Neurological Manifestation of Sacral Tumors. Neurosurg Focus (2003) 15(2):E1. doi: 10.3171/foc.2003.15.2.1 15350032

[10] Fourney DR Frangou EM Ryken TC Dipaola CP Shaffrey CI Berven SH. Spinal Instability Neoplastic Score: An Analysis of Reliability and Validity From the Spine Oncology Study Group. J Clin Oncol (2011) 29(22):3072–7. doi: 10.1200/jco.2010.34.3897 21709187

[11] Takeshima N Miyakawa H Okuda K Hattori S Hagiwara S Takatani J. Evaluation of the Therapeutic Results of Epiduroscopic Adhesiolysis for Failed Back Surgery Syndrome. Br J Anaesth (2009) 102(3):400–7. doi: 10.1093/bja/aen383 19164308

[12] Agha RA Sohrabi C Mathew G Franchi T Kerwan A O’Neill N. The PROCESS 2020 Guideline: Updating Consensus Preferred Reporting Of CasESeries in Surgery (PROCESS) Guidelines. Int J Surg (2020) 84:231–5. doi: 10.1016/j.ijsu.2020.11.005 33189880

[13] Gosling T Pichlmaier MA Langer F Krettek C Hufner T. Two-Stage Multilevel En Bloc Spondylectomy With Resection and Replacement of the Aorta. Eur Spine J (2013) 22 Suppl 3:S363–8. doi: 10.1007/s00586-012-2471-0 PMC3641252 22972602

[14] Somasundaram A Wicks RT Lata AL Qasem SA Hsu W. En Bloc Spondylectomy for Primary Malignant Fibrous Histiocytoma of the Thoracic Spine With Aortic Involvement: Case Report. J Neurosurg Spine (2015) 22(4):399–405. doi: 10.3171/2014.9.spine14155 25658464

[15] Pilger A Tsilimparis N Bockhorn M Trepel M Dreimann M. Combined Modified En Bloc Corpectomy With Replacement of the Aorta in Curative Interdisciplinary Treatment of a Large Osteosarcoma Infiltrating the Aorta. Eur Spine J (2016) 25 Suppl 1:58–62. doi: 10.1007/s00586-015-4079-7 26112246

[16] Kao TH Shen CC Chen CC Kwan PH. “Primary” Benign Retroperitoneal and Intraspinal Dumbbell-Shaped Cystic Teratoma: Case Report. Spine (Phila Pa 1976) (2005) 30(15):E439–43. doi: 10.1097/01.brs.0000172227.46437.63 16094263

[17] Zoccali C Marolda G Di Francesco A Favale L Salducca N Biagini R. Pelvic Sacral and Hemi Lumbar Spine Resection of Low Grade Pelvic Chondrosarcoma: A Multistage Procedure Involving Vascular Bypass, Spine Fixation and Vascular Exclusion. Orthop Traumatol Surg Res (2013) 99(7):875–9. doi: 10.1016/j.otsr.2013.05.007 24074762

[18] Tonomura H Hatta Y Nagae M Takatori R Kubo T. Posterior Resection of Fifth Lumbar Giant Schwannoma Combined With a Recapping Transiliac Approach: Case Report and Technical Note. Eur J Orthop Surg Traumatol (2018) 28(6):1209–14. doi: 10.1007/s00590-018-2178-y 29536189

[19] Mirza AK Alvi MA Naylor RM Kerezoudis P Krauss WE Clarke MJ. Management of Major Vascular Injury During Pedicle Screw Instrumentation of Thoracolumbar Spine. Clin Neurol Neurosurg (2017) 163:53–9. doi: 10.1016/j.clineuro.2017.10.011 29073499

[20] Schwender JD Casnellie MT Perra JH Transfeldt EE Pinto MR Denis F. Perioperative Complications in Revision Anterior Lumbar Spine Surgery: Incidence and Risk Factors. Spine (Phila Pa 1976) (2009) 34(1):87–90. doi: 10.1097/BRS.0b013e3181918ad0 19127166

[21] Riaz H Alansari SA Khan MS Riaz T Raza S Luni FK. Safety and Use of Anticoagulation After Aortic Valve Replacement With Bioprostheses: A Meta-Analysis. Circ Cardiovasc Qual Outcomes (2016) 9(3):294–302. doi: 10.1161/circoutcomes.115.002696 27166205 PMC5124764

[22] Khan MF Burks SS Al-Khayat H Levi AD. The Effect of Steroids on the Incidence of Gastrointestinal Hemorrhage After Spinal Cord Injury: A Case-Controlled Study. Spinal Cord (2014) 52(1):58–60. doi: 10.1038/sc.2013.122 24145687

[23] Fineberg SJ Kurd MF Patel AA Singh K. Incidence and Risk Factors for Gastrointestinal Hemorrhage After Lumbar Fusion. Spine (Phila Pa 1976) (2013) 38(18):1584–9. doi: 10.1097/BRS.0b013e318298768d 23632338

[24] Voin V Kirkpatrick C Alonso F Rustagi T Sanders FH Drazin D. Lateral Transpsoas Approach to the Lumbar Spine and Relationship of the Ureter: Anatomic Study With Application to Minimizing Complications. World Neurosurg (2017) 104:674–8. doi: 10.1016/j.wneu.2017.05.062 28532911

[25] Moreno-Alarcon C Lopez-Cubillana P Lopez-Gonzalez PA Prieto-Gonzalez A Ruiz-Morcillo JC Olarte-Barragan EH. Lich-Gregoir Technique and Routine Use of Double J Catheter as the Best Combination to Avoid Urinary Complications in Kidney Transplantation. Transplant Proc (2014) 46(1):167–9. doi: 10.1016/j.transproceed.2013.12.002 24507045

[26] Cavalli AC Tambara Filho R Slongo LE Cavalli RC Rocha LC. The Use of Double-J Catheter Decreases Complications of Retroperitoneoscopic Ureterolithotomy. Rev Col Bras Cir (2012) 39(2):112–8. doi: 10.1590/S0100-69912012000200006 22664517

[27] Golubovsky JL Ilyas H Chen J Tanenbaum JE Mroz TE Steinmetz MP. Risk Factors and Associated Complications for Postoperative Urinary Retention After Lumbar Surgery for Lumbar Spinal Stenosis. Spine J (2018) 18(9):1533–9. doi: 10.1016/j.spinee.2018.01.022 29447854

[28] Gandhi SD Patel SA Maltenfort M Anderson DG Vaccaro AR Albert TJ. Patient and Surgical Factors Associated With Postoperative Urinary Retention After Lumbar Spine Surgery. Spine (Phila Pa 1976) (2014) 39(22):1905–9. doi: 10.1097/brs.0000000000000572 25299169

